# A nationwide population-based study of the inflammatory bowel diseases between 1998 and 2008 in Taiwan

**DOI:** 10.1186/1471-230X-13-166

**Published:** 2013-12-06

**Authors:** Shu-Chen Wei, Meng-Hung Lin, Chien-Chih Tung, Meng-Tzu Weng, Jen-Shin Kuo, Ming-Jium Shieh, Cheng-Yi Wang, Wen-Chao Ho, Jau-Min Wong, Pau-Chung Chen

**Affiliations:** 1 Department of Internal Medicine, National Taiwan University Hospital and College of Medicine, 7 Chung Shan South Road, Taipei, Taiwan; 2 Department of Public Health, College of Public Health, China Medical University, Taichung, Taiwan; 3 Department of Integrated Diagnostics and Therapeutics, National Taiwan University Hospital and College of Medicine, Taipei, Taiwan; 4 Department of Internal Medicine, Far Eastern Memorial Hospital, New Taipei, Taiwan; 5 Department of Internal Medicine, Kang-Ning General Hospital, Taipei, Taiwan; 6 Department of Oncology, National Taiwan University Hospital and College of Medicine, Taipei, Taiwan; 7 Institute of Occupational Medicine and Industrial Hygiene, National Taiwan University College of Public Health, 17 XuZhou Road, Taipei, Taiwan; 8 Department of Public Health, National Taiwan University College of Public Health, Taipei, Taiwan; 9 Department of Environmental and Occupational Medicine, National Taiwan University Hospital and College of Medicine, Taipei, Taiwan

**Keywords:** Crohn’s disease, Ulcerative colitis, Incidence, Prevalence, Taiwan, Nationwide

## Abstract

**Background:**

The incidence of the inflammatory bowel diseases (IBD), ulcerative colitis (UC) and Crohn’s disease (CD), has been increasing in Asia. We probed the nationwide registered database to assess the incidence, prevalence, gender distribution, age of diagnosis and the survival status of IBD patients in Taiwan.

**Methods:**

A retrospective study was conducted to analyze the registered database compiled by the National Health Insurance provided by the Department of Health, Taiwan, from January 1998 through December 2008.

**Results:**

A total of 1591 IBD patients were registered from 1998 to 2008 in Taiwan (CD: 385; UC: 1206). The incidence of CD increased from 0.19/100,000 in 1998 to 0.24/100,000 in 2008. The incidence of UC increased from 0.61/100,000 in 1998 to 0.94/100,000 in 2008. The prevalence of CD increased from 0.19/100,000 in 1998 to 1.78/100,000 in 2008. The prevalence of UC increased from 0.61/100,000 in 1998 to 7.62/100,000 in 2008. Male to female ratio for CD was 2.22 and 1.64 for UC. Age of registered for CD was predominantly between 20 to 39, and for UC between 30 to 49 years of age. The standardized mortality ratio (95% CI) was 4.97 (3.72–6.63) for CD and 1.78 (1.46–2.17) for UC, from 1998 to 2008 in Taiwan.

**Conclusions:**

Using the Taiwan nationwide database for IBD, the incidence and prevalence of IBD in Taiwan significantly increased from 1998 to 2008. The mortality rate was higher for CD patients than UC patients, and both were higher than the general population.

## Background

The inflammatory bowel diseases (IBDs) Crohn’s disease (CD) and ulcerative colitis (UC) are common causes of chronic gastrointestinal disease in the developed world. Initially regarded as a Western lifestyle disease, inflammatory bowel disease (IBD) is increasing in both incidence and prevalence in many parts of the Asia Pacific area [1-8]. The reason for this increasing trend has not been established but is most likely related to environment factors, including improved hygiene and “Westernization” of diet [9].

Based on a hospital (National Taiwan University Hospital)-based analysis, we previously showed that CD as well as UC has increased in both incidence and prevalence in Taiwan [10-12]. The National Taiwan University Hospital is a tertiary referral center in Taiwan; with the first case of ulcerative colitis being diagnosed in 1969. The previous reports offered a detailed disease phenotypic analysis with a long follow-up period (from 1989 to 2008). However, the exact condition of the general incidence and prevalence of CD and UC in Taiwan still needs a nationwide-based collection of data for a more representative and comprehensive analysis.

In Taiwan, the National Health Insurance provided by the Department of Health, Executive Yuan of Taiwan government was launched in 1995. Almost 99% of the residents of Taiwan subscribe to this program for their health insurance. Since 1997, CD and UC, due to their potential for repetitive admissions and the need for chronic, careful caring, are registered as catastrophic illnesses. When a patient’s ailment is diagnosed by a physician as a “catastrophic illness” under Department of Health guidelines, the patient can submit related information and apply for a catastrophic illness certificate/registration. For IBD, the application includes the clinical diagnosis, pathological report as well as the possible image studies to be formally reviewed to validate and register the patient’s diagnosis of CD or UC, as well as for excluding intestinal tuberculosis. In this study, we used this nationwide-based data set to reveal the incidence, prevalence, age/gender distribution and the mortality rate of IBD patients in Taiwan.

## Methods

### Data source and ethical considerations

The nationwide population-based Taiwanese study of IBD was compiled between January 1998 and December 2008. Data was obtained from the Taiwan National Health Insurance (NHI) research database, which has been routinely collected by the National Health Research Institute (NHRI). These high quality databases have previously been used for epidemiologic research, information on prescription use, diagnoses, and hospitalizations [13-15]. With strict confidentiality guidelines being closely followed in accordance with personal electronic data protection regulations; the NHRI anonymized and maintained the NHI reimbursement data as files suitable for research. In addition, this study was approved by the Ethics Review Board at the National Taiwan University Hospital.

### Patient identification

We used the diagnostic code (The International Classification of Diseases, Ninth Revision, Clinical Modification 2001 edition) to retrieve the IBD patients (UC: 556.XX; CD: 555.XX) from the catastrophic illness registration database. For each patient, medical records were collected in the NHI claim database, including date of admission, date of discharge, dates of visits, clinical diagnosis, prescription and total expenditure. The databases also contained patient information, including sex, date of birth and date of death.

### Statistical analyses

The characteristics of IBD patients, including the year of diagnosis, sex ratio and age distribution, were described in this study. The annual incidence and prevalence were defined as the number of newly diagnosed patients and the number of patients with IBD per 100,000 persons per year, respectively, namely, the crude incidence and prevalence. We used the Kaplan-Meier method to estimate IBD cumulative survival rate over an eleven year follow-up period. To compare with the general population of Taiwan, the standardized mortality ratio and 95% confidence interval were calculated based on the Poisson assumption. Furthermore, the direct mortality rate was also standardized to the WHO 2000 standard population using 5-year age groups per 1,000 person-years. All statistical analyses were performed with the SAS version 9.3 (SAS Institute, Cary, NC, USA). A *P* value of less than 0.05 was set to declare statistical significance.

## Results

**Figure 1:**
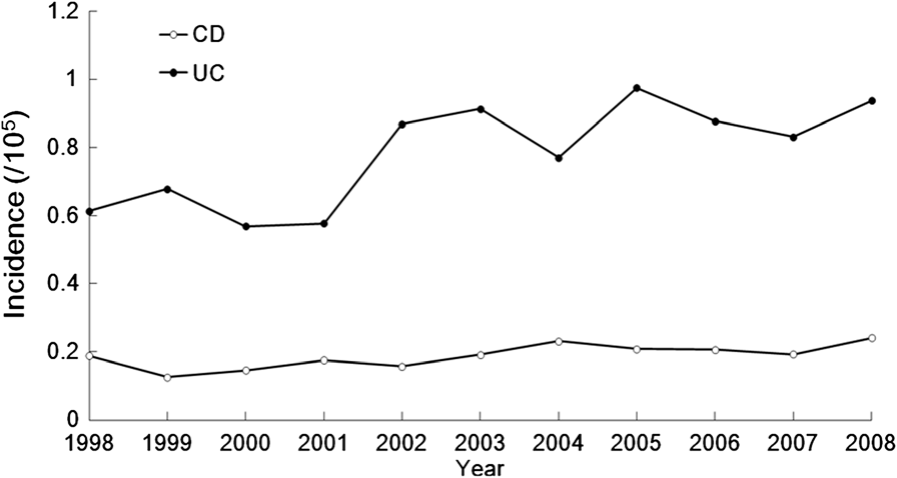
Incidence of IBD from 1998–2008 in Taiwan.

A total of 1,591 IBD patients were registered from 1998 to 2008 in Taiwan. Among them, 385 were diagnosed with CD and 1,206 with UC. As shown in Figure 1, the incidence of CD increased from 0.19/100,000 in 1998 to 0.24/100,000 in 2008. The incidence of UC increased from 0.61/100,000 in 1998 to 0.94/100,000 in 2008. When this period was arbitrarily divided into the first stage as 1998 to 2003 and second stage from 2004 to 2008, the mean incidence for CD in the first stage was 0.16/100,000 and 0.22/100,000 for the second stage; the incidence of CD increased significantly from the first stage to the second stage (p = 0.005). For UC, the mean incidence in the first stage was 0.70/100,000 and 0.88/100,000 for the second stage; the incidence of UC increased also significantly from the first stage to the second stage (p = 0.047).

**Figure 2:**
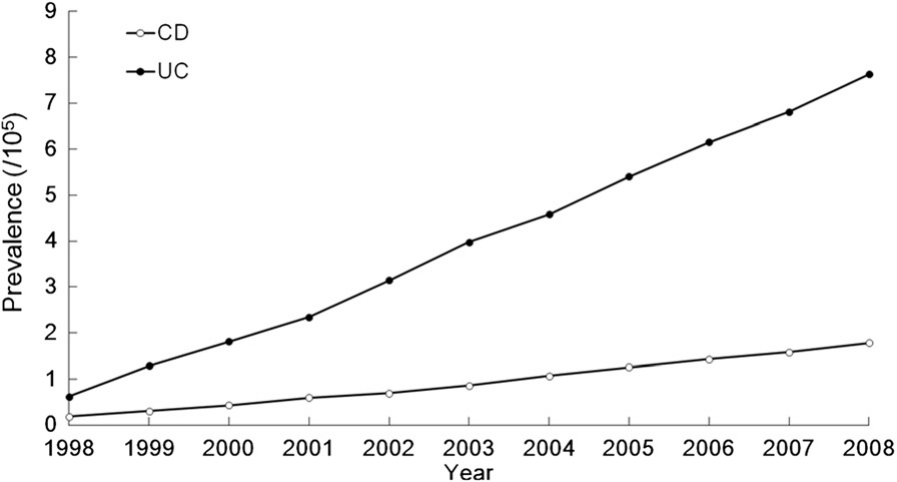
Prevalence of IBD from 1998–2008 in Taiwan.

As shown in Figure 2, the prevalence of CD increased from 0.19/100,000 in 1998 to 1.78/100,000 in 2008. The prevalence of UC increased from 0.62/100,000 in 1998 to 7.62/100,000 in 2008. The mean prevalence for CD in the first stage was 0.51/100,000 and 1.42/100,000 for the second stage; the prevalence of CD increased significantly from the first stage to the second stage (p = 0.0003). For UC, the mean prevalence in the first stage was 2.2/100,000 and 6.12/100,000 for the second stage; the prevalence of UC increased also significantly from the first stage to the second stage (p = 0.0005).

**Figure 3:**
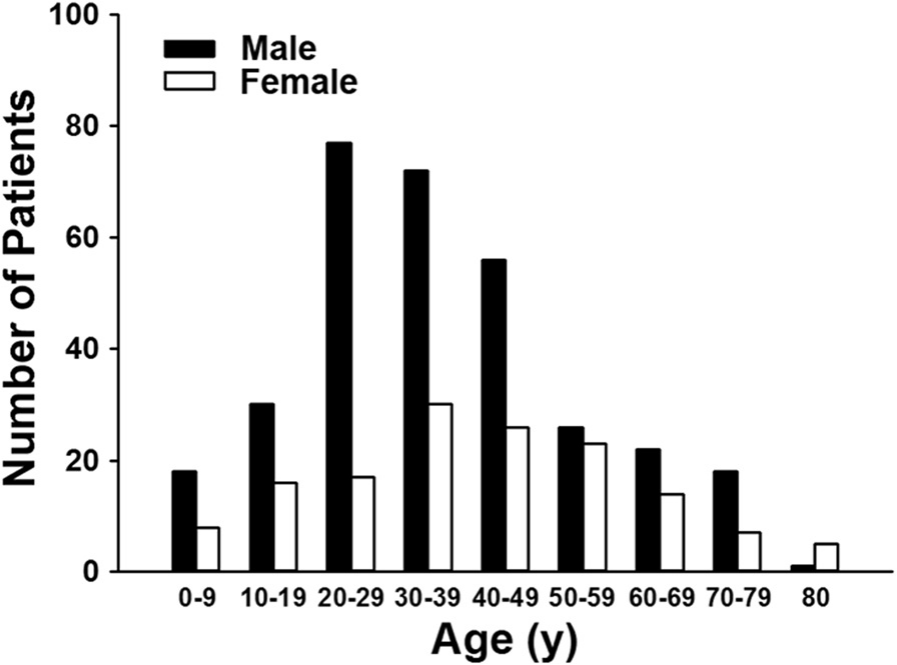
Age and sex distribution of Crohn’s disease patients from 1998–2008 in Taiwan.

**Figure 4:**
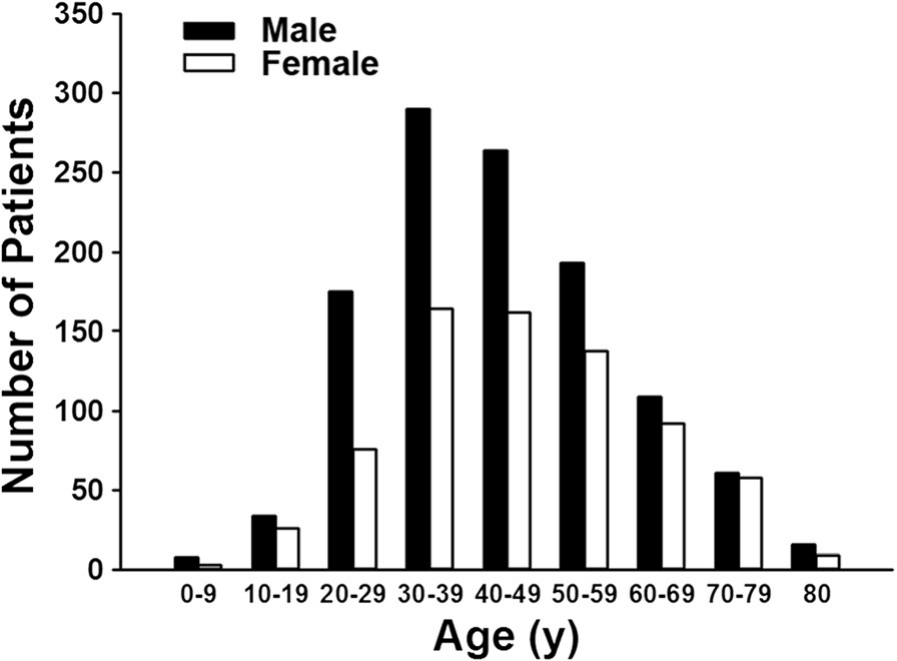
Age and sex distribution of ulcerative colitis patients from 1998–2008 in Taiwan.

**Figure 5:**
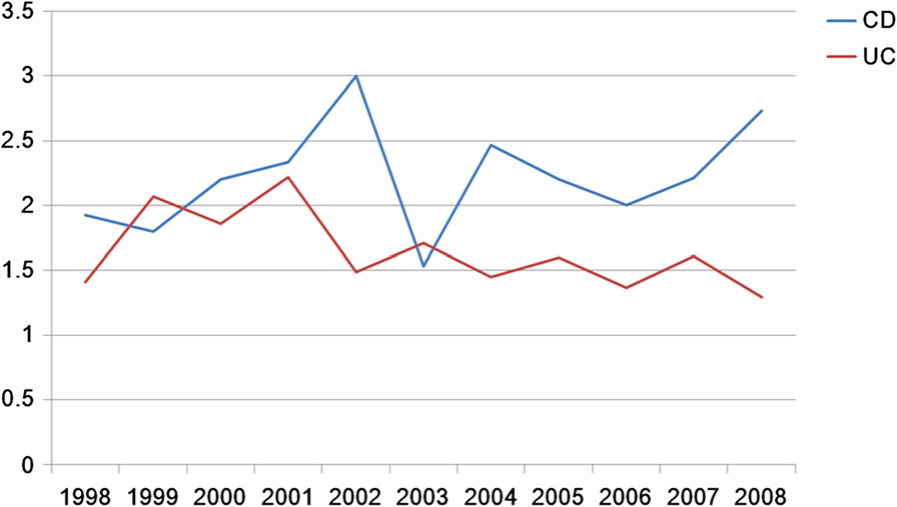
Gender ratio (male to female) of IBD patients in Taiwan from 1998 to 2008.

The year of diagnosis and sex-specific number of patients with CD and UC in Taiwan from 1998 to 2008 are summarized in Table 1. Sex- and age-specific number of patients registered with CD are summarized in Table 2, and for UC in Table 3. The age of registration for CD patients was primarily between 20 to 39 (Figure 3), with the mean and standard deviation of 37.8 and 18.8 years of age, respectively. The age of registration for UC was mostly between 30 to 49 years old (Figure 4), with the mean and standard deviation of 44.5 and 15.8 years old, respectively. Male to female ratios for CD was 2.22 and 1.64 for UC. These ratios reflect a male predominant pattern in Taiwan for CD over UC (p = 0.01) (Figure 5).

**Table 1:** Year of diagnosis and sex-specific number of patients with Crohn’s disease and ulcerative colitis in Taiwan, 1998-2008

**Year of diagnosis**	**Male**	**Female**	**Total**	**Male to female ratio**
Crohn’s disease				
1998	25	13	38	1.9
1999	18	10	28	1.8
2000	22	10	32	2.2
2001	28	12	40	2.3
2002	27	9	36	3
2003	26	17	43	1.5
2004	37	15	52	2.5
2005	33	15	48	2.2
2006	32	16	48	2
2007	31	14	45	2.2
2008	41	15	56	2.7
Ulcerative colitis				
1998	69	49	118	1.4
1999	91	44	135	2.1
2000	78	42	120	1.9
2001	82	37	119	2.2
2002	113	76	189	1.5
2003	123	72	195	1.7
2004	100	69	169	1.4
2005	136	85	221	1.6
2006	116	85	201	1.4
2007	119	74	193	1.6
2008	123	95	218	1.3

**Table 2:** Sex- and age-specific number of patients registered as Crohn’s disease in Taiwan, 1998-2008

**Age group**	**Male**	**Female**	**Total**
0–9	18	8	26
10–19	30	16	46
20–29	77	17	94
30–39	72	30	102
40–49	56	26	82
50–59	26	23	49
60–69	22	14	36
70–79	18	7	25
≥80	1	5	6

**Table 3:** Sex- and age-specific number of patients registered as ulcerative colitis in Taiwan, 1998-2008

**Age group**	**Male**	**Female**	**Total**
0–9	8	3	11
10–19	34	26	60
20–29	175	76	251
30–39	290	164	454
40–49	264	162	426
50–59	193	138	331
60–69	109	92	201
70–79	61	58	119
≥80	16	9	25

**Figure 6:**
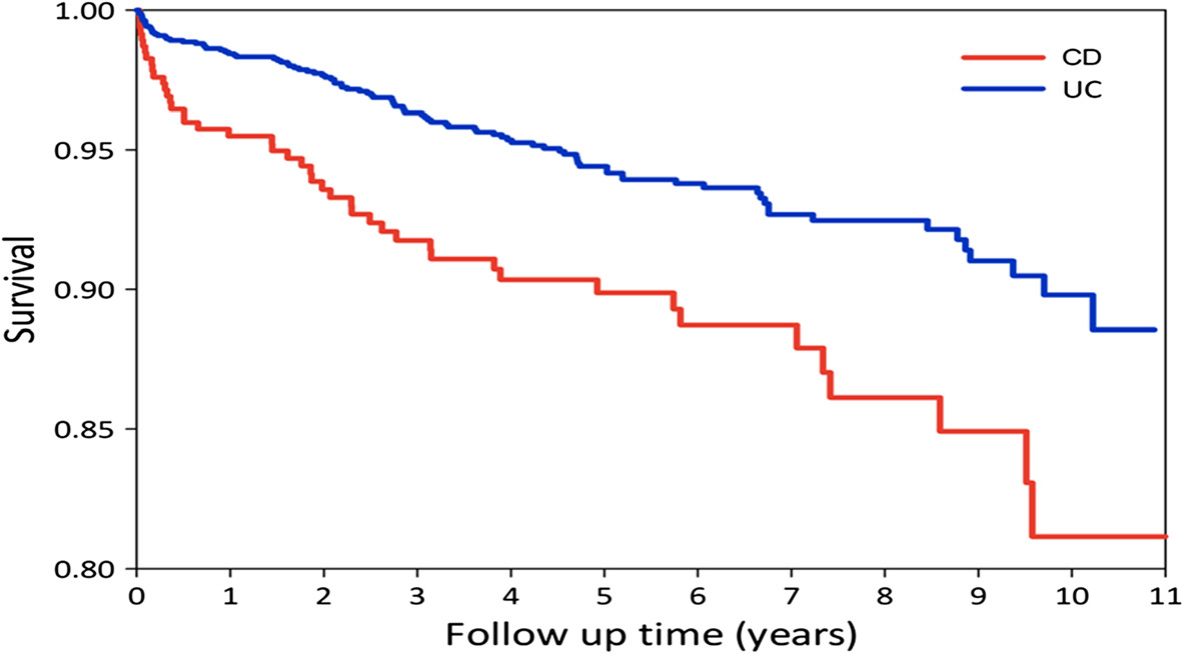
Survival rate of IBD patients in Taiwan from 1998 to 2008.

As shown in Figure 6, the survival status of CD patients after registration for one year, 5 years and 10 years were: 96%, 90% and 82%, respectively. For UC patients, the survival status after registration for one year, 5 years and 10 years were: 99%, 95% and 90%, respectively. There was a statistical difference between the survival status of UC and CD patients from 1998 to 2008 in Taiwan (p = 0.001). The standardized mortality ratio (95% CI) was 4.97 (3.72–6.63) and the standardized mortality rate (per 1,000 person-years) was 29.7 for CD; and the standardized mortality ratio (95% CI) was 1.78 (1.46–2.17) with the standardized mortality rate (per 1,000 person-years) of 9.62 for UC from 1998 to 2008 in Taiwan.

## Discussion

Increased incidence and prevalence of IBD in Asia have been reported [1,3-12]. This information is mostly based on hospital-based data except for a report from Japan where there is a nationwide registration system [16]. By using a nationwide registration system in Taiwan, we confirmed this phenomenon by adding a second nationwide-based data analysis in Asia, which confirmed that the incidence and prevalence of IBD increased from 1998 to 2008 in Taiwan. From our experience, the true IBD patient number should be more than the registered number as there are always IBD patients who have not yet registered or who have not passed the registration process, therefore, there is no way to know the real number. The bottom line is that we have at least the number of cases reported in this study with a confirmed diagnosis of IBD and the number of cases increased significantly over the past years in Taiwan. Although the incidence (CD: 0.24/100,000; UC: 0.94/100,000, in 2008) and prevalence (CD: 1.78/100,000; UC: 7.62/100,000, in 2008) increased in Taiwan, they were still lower than the reports from Japan (prevalence: approximately 21/100,000 for CD and 63.6/100,000 for UC), Korea (prevalence: approximately 11/100,000 for CD and 30.9/100,000 for UC), and much lower than those from Western Countries (prevalence: approximately 200/100,000 for CD and 400/100,000 for UC) [1,9,16]. And according to the recently pusblised large scale population-based epidemiologic study across nine countries in Asia-Pacific showing geographic variability in disease incidence even within Asia [17]. Taiwan appeared to have disease incidence that is lower than Hong Kong, some parts of mainland China, and Macau, and relatively similar to that of Malaysia and Sri Lanka, but higher than those of Thailand and Indonesia. One may speculate that different degrees of urbanization/socioeconomic status may play a part in the variation.

The mean age of patients registered with CD was 37.8 and for UC was 44.5 years of age. We previously have shown that, based on a referral center in Taiwan, the mean age of diagnosis for CD was 30.5 and for UC was 36 in the National Taiwan University Hospital [11,12]. This difference might be related to the lag between diagnosis and registration, as well as the possible gap between the IBD diagnosis efficiency between an experienced center and the other general hospitals. Nonetheless, the mean age for CD and UC registered patients was still within the range reported from other countries in Asia [16].

Considering the male to female ratio, in the current study we found the ratio for CD was 2.22 and for UC was 1.64, which was consistent with our previous results which showed 1.82 for CD and 1.35 for UC [11,12]. Both diseases showed a male predominant pattern, especially significant for CD. This observation was consistent with the reports from the other Asian countries, most of them revealed a male predominant prevalence. Interestingly, this differed from reports from Western countries [16,18].

The survival status, or vice versa, the mortality rate from this report was quite different from previous reports, even when compared to our previous results based on a referral center follow-up study which showed the accumulated mortality rate for CD to be 2.7% (3 of 110) and 1.72% for UC (7 of 406) from 1988 to 2008. In this study, the survival rate of CD patients after the registration for one year, 5 years and 10 years were: 96%, 90% and 82%, respectively. For UC patients, the survival status after the registration for one year, 5 years and 10 years were: 99%, 95% and 90%, respectively. There was a statistical difference of the survival status between UC and CD patients from 1998 to 2008 in Taiwan (p = 0.001). When compared with the general population, the standardized mortality ratio (95% CI) was 4.97 (3.72–6.63) and the standardized mortality rate (per 1,000 person-years) was 29.7 for CD; the standardized mortality ratio (95% CI) was 1.78 (1.46–2.17) with the standardized mortality rate (per 1,000 person-years) as 9.62 for UC from 1998 to 2008 in Taiwan. Both were higher than the reports from Japan as well as from most Western countries, with the standardized mortality ratio for CD around 1.5 fold the general population. The mortality rate of CD patients was slightly higher than the general population and the mortality rates for UC patients, either from Japan or from Western countries were mostly the same as the general population [19-31].

The higher mortality shown in this study was unexpected. However, as the data was anonymized, only the date of death but not the cause of death nor comorbidity could be traced from this data set. The registered data only provided the diagnosis, no phenotypic information was included. Therefore, we were not able to know whether the higher mortality resulted from the severity of the disease or not. The ages of diagnosis in this cohort were comparable to the reports from other Asian countries [1,17,30], age did not seem to be able to explain the higher mortality. However, when comparing the results from a referral center (National Taiwan University Hospital, for example) with the nationwide population-base, patients tended to be diagnosed at earlier ages and with less mortality when seen in a referral center. This result might reflect the low awareness of IBD in the general practice, since previously in Taiwan,IBD was a relatively low prevalence disease. Experience diagnosing and treating IBD patients when they presented outside the referral center (NTUH, for example) was lagging. Therefore, since 2010, we have started to emphasize the increasing trend of IBD as well as an IBD awareness education program. Hopefully in the future, we can improve the awareness, the quality of care, and the outcome of IBD patients in Taiwan. Our experience might also afford other countries where IBD is low in prevalence, to modify their quality of care in treating IBD patients which should translate into improving the prognosis of IBD patients.

## Conclusion

Using the Taiwan nationwide database for IBD, the incidence and prevalence of IBD in Taiwan significantly increased from 1998 to 2008.

The mortality rate was higher for CD patients than UC patients, and both were higher than the general population. After our promotion of the disease awareness, we hope the outcome of IBD patients in Taiwan could be improved in the future.

## Competing interests

The authors declare that they have no competing interests.

## Authors’ contributions

SCW, CCT, MTW, JSK, MJS, CYW, JMW: study desing, data collection, manuscript preparation. MHL, WCH, PCC: statistics analysis and manucript preparation. All authors read and approved the final manuscript.

## Pre-publication history

The pre-publication history for this paper can be accessed here:

http://www.biomedcentral.com/1471-230X/13/166/prepub
